# Permanent pacemaker implantation for atrioventricular block secondary to acute pancreatitis in a patient affected by panhypopituitarism

**DOI:** 10.1186/s43044-024-00590-3

**Published:** 2024-12-12

**Authors:** Jacopo Giovacchini, Silvia Menale, Irene Merilli, Valentina Scheggi

**Affiliations:** 1 Division of Cardiovascular and Perioperative Medicine, Careggi University Hospital, Largo Brambilla 3, 50134 Florence, Italy; 2 Division of General Cardiology, Department of Cardiothoracovascular Medicine, Careggi University Hospital, Largo Brambilla 3, 50134 Florence, Italy; 3 Department of Experimental and Clinical Medicine, University of Florence, Largo Brambilla 3, 50134 Florence, Italy

**Keywords:** Panhypopituitarism, Acute pancreatitis, Atrioventricular block, Pacemaker, Case report

## Abstract

**Background:**

Hypopituitarism may trigger the development of acute pancreatitis (AP) through multiple mechanisms. AP may alter normal intracardiac conduction leading to an atrioventricular block. Due to the lack of similar cases, the correct timing and indication for pacemaker implantation in such a setting are unknown.

**Case presentation:**

A 22-year-old woman with a history of sub-total excision of frontal astrocytoma with residual panhypopituitarism in replacement therapy was admitted to the emergency department with AP and peripancreatic necrosis, complicated by hypotension, sinus bradycardia with 2:1 atrioventricular block, and severe acute respiratory distress syndrome deserving intubation and mechanical ventilation. During the in-hospital course, the patient developed a systemic inflammatory response syndrome and acute kidney failure and was treated with intravenous dopamine, diuretics, and liquids. While she gradually recovered, advanced atrioventricular block persisted after the resolution of AP; therefore, a permanent pacemaker was implanted. During the follow-up, appropriate device interventions were detected.

**Conclusions:**

No other cases of high-grade atrioventricular block in panhypopituitarism-induced AP have been reported in the literature. Our case suggests a pacemaker is necessary if the atrioventricular block does not recover with AP resolution. Further evidence is required to improve the management of rhythm disturbances in hypopituitarism patients who develop AP.

## Background

Acute pancreatitis (AP) may derive from a mechanical issue (i.e. gallstones, pancreatic duct obstructions, traumas, iatrogenic injuries, etc.), a toxic/metabolic insult (i.e. alcohol, hyperlipidemias, drugs, etc.), or other factors (i.e. ischemia, infections, autoimmunity, hereditary) [1]. Hypopituitarism can also lead to AP due to metabolic and endocrine alterations.

During AP, abnormal electrocardiographic (ECG) findings have been observed, including sinus bradycardia (SB), intraventricular conduction defects, atrioventricular block (AVB), and T-wave or ST-segment changes. They may be explained by a direct cardio-toxic effect of inflammatory cytokines and proteolytic enzymes, cardio-biliary reflex, coronary artery spasm, and metabolic alterations with electrolyte abnormalities [2–4].

We present a case of advanced AVB that required a permanent pacemaker (PM) implantation following an episode of AP in a patient with hypopituitarism.

## Case presentation

**Fig. 1:**
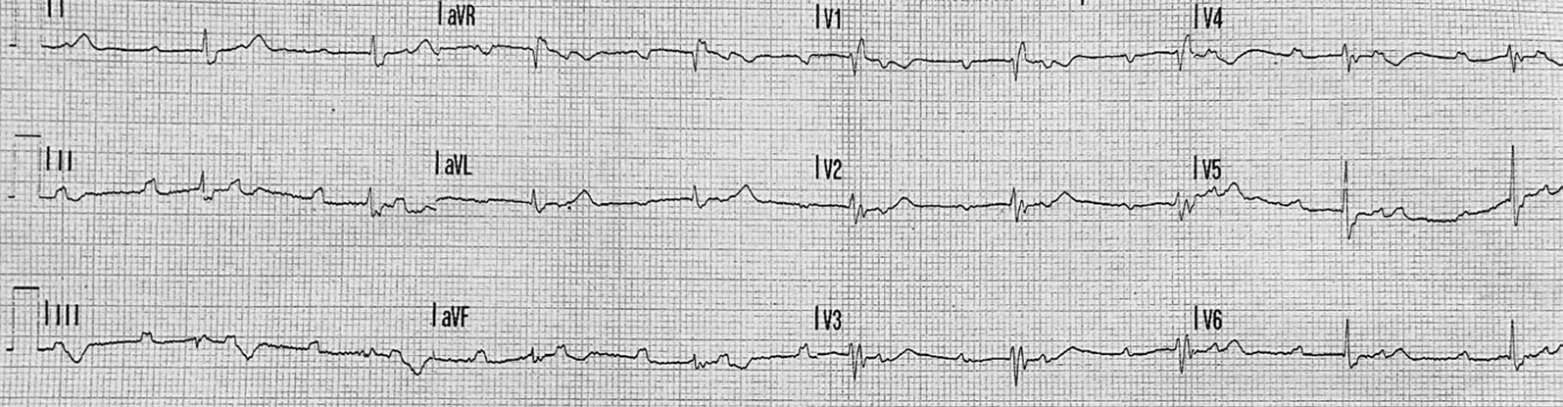
ECG on admission, showing sinus rhythm 55 b.p.m. with 2:1 AVB

**Fig. 2:**
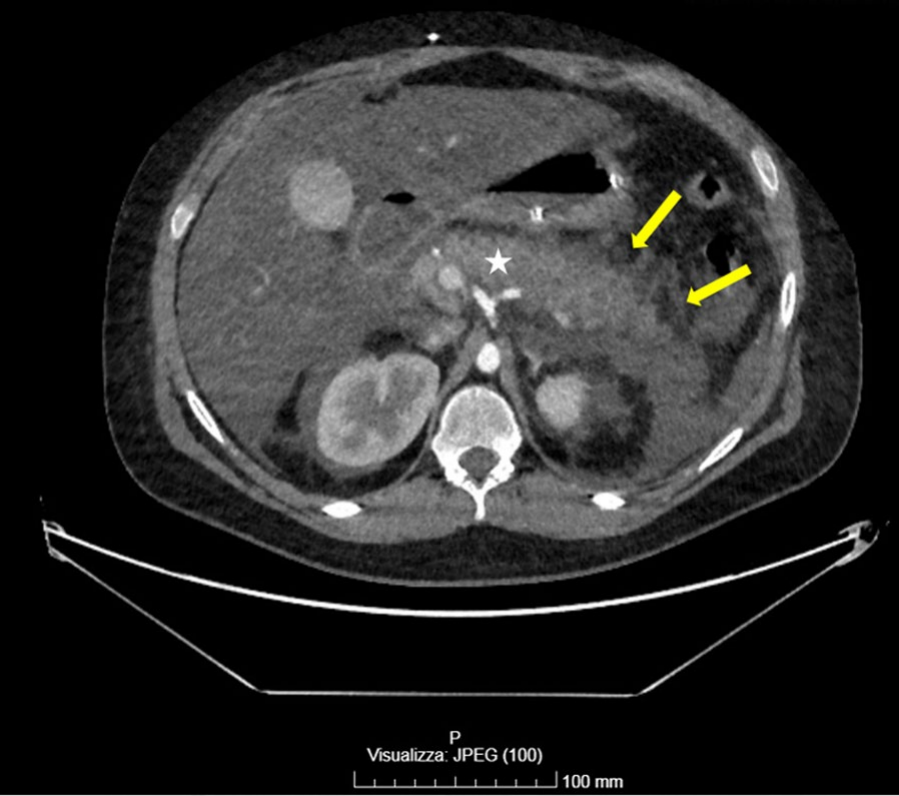
Abdominal contrast-enhanced CT scan, arterial phase, showing a globular pancreas increased in volume (white ✩) along with peripancreatic necrotic areas (yellow arrows)

A 22-year-old woman with previous sub-total excision of frontal astrocytoma and residual panhypopituitarism in replacement therapy was admitted to the emergency department due to resting dyspnoea, nausea, vomiting, and diffuse abdominal pain. Upon clinical examination, the patient was hypotensive with SB (55 beats per minute—b.p.m.) as a result of a 2:1 AVB at ECG (Fig. 1). Additionally, she was dyspnoeic with type II respiratory insufficiency. Echocardiography yielded normal results. Blood tests revealed elevated serum lipase levels (590 U/L, upper reference value 160 U/L). A contrast-enhanced total body computed tomography (CT) scan demonstrated a swollen and globular pancreas with loss of typical lobulation, and diffuse fluid collection within the periglandular, periduodenal, and pararenal spaces (Fig. 2). According to the Atlanta classification [5], acute interstitial oedematous pancreatitis (AIP) was diagnosed.

The patient was admitted to the intensive care unit. On admission, she was hypotensive, bradycardic, with severe acute respiratory distress syndrome. Therefore, she was intubated and mechanically ventilated. An adrenal crisis secondary to AIP was diagnosed due to extremely low serum cortisol levels (14 pg/mL). Intravenous hydrocortisone was administered (50 mg i.v. every 6 h, then de-escalated). Because of acute kidney injury and oligo-anuria, her desmopressin maintenance therapy was discontinued, and crystalloid fluids and intravenous dopamine infusion were instituted to address hypotension and bradyarrhythmia. Diuretic therapy was slowly up-titrated. A repeated abdominal CT scan revealed a significant increase in peripancreatic and retroperitoneal fluid, with adipose-like areolae consistent with necrotizing pancreatitis (peripancreatic necrosis only phenotype, according to the Atlanta classification [5]). Parenteral and slow trophic enteral nutrition were carried on. The patient experienced systemic inflammatory response syndrome (SIRS) (high fever, marked increase in C-reactive protein and leucocytosis) with negative blood cultures. Abundant intravenous fluid replacement was required.

As renal function improved, a marked polyureic state along with hypernatremia ensued; as a result, diuretics were discontinued and desmopressin was re-introduced (50 µg/mL, 2 puffs *ter in die*). Levothyroxine dosage was augmented because of subclinical hypothyroidism.

The patient slowly improved, eventually being weaned by the respiratory and circulatory support. Blood tests indicated restoration of pancreatic and adrenal function (peak lipase levels 2231 U/L). Oral feeding was resumed. Both steroid and desmopressin therapy were de-escalated (i.e. switched to 1.5 tablet *die* of oral cortisone acetate 25 mg and 1 tablet *ter in die* of desmopressin 120 µg, respectively). Subsequent abdominal CT scans demonstrated a gradual decrease in pancreatic hypodense lesions and peripancreatic and intra-abdominal effusions, without signs of superinfection.

We suspected that the patient’s hypopituitarism could have primed AP, as no alterations in bile ducts were identified, negative viral antibodies and blood cultures excluded an infective cause, and the autoimmune panel was unsuggestive. Additionally, her serum calcium was normal and her medication history did not include drugs harmful to the pancreas.

**Fig. 3:**
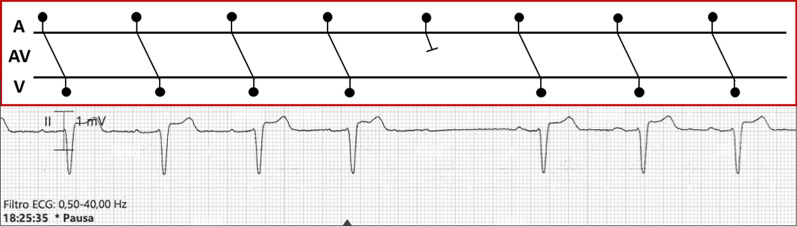
Patient’s continuous ECG monitoring with Lewis-ladder diagram: sinus rhythm with a diurnal Mobitz type II AVB episode

**Fig. 4:**
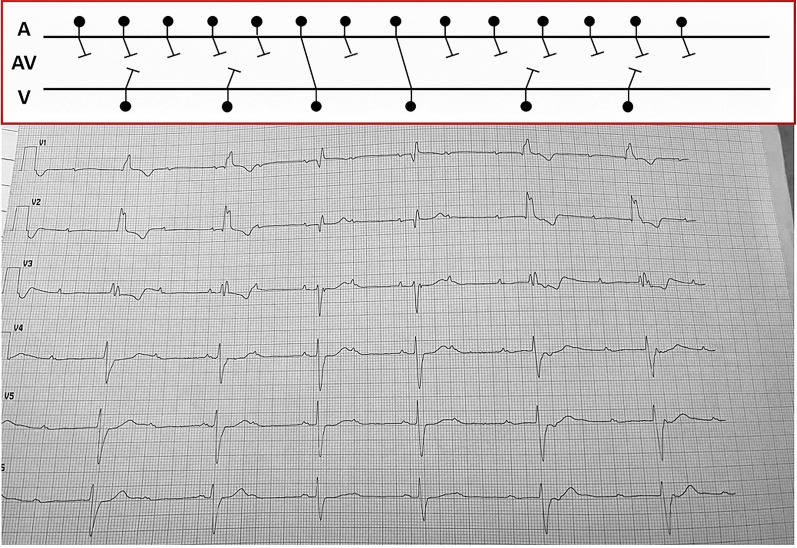
Patient’s ECG with Lewis-ladder diagram: sinus rhythm 37 b.p.m., advanced AVB and AV dissociation with ventricular escape rhythm (wide QRS with right bundle branch morphology, different from captured-QRS ones)

Heart rhythm was monitored throughout the hospital stay, with the persistence of sinus rhythm along with phases of both Mobitz type II AVB (Fig. 3), 2:1 atrioventricular (AV) conduction, and advanced AVB with AV dissociation (Fig. 4). Therefore, as no reversible causes could be identified (i.e. normal electrolytes and thyroid function) and because the patient was apyretic with negative serial blood cultures, a permanent bicameral PM was implanted and programmed in DDD-40 b.p.m.

The patient was discharged after 24 days of hospitalisation. During follow-up visits, the PM interrogation showed appropriate device interventions.

## Discussion

Our patient had a history of surgically resected frontal astrocytoma with residual panhypopituitarism. Astrocytoma is a rare brain tumour responsible for a wide spectrum of endocrine and metabolic disorders [6]; furthermore, when radical surgery is performed, it may lead to iatrogenic hypopituitarism [7].

Hypopituitarism can precipitate AP by affecting circulating lipoproteins, disrupting satiety and hunger control, and impairing sensitivity to the leptin–insulin–ghrelin axis [8].

Having excluded all other possible causes, we concluded that the patient’s hypopituitarism could have provoked the AP episode. Then, advanced AVB occurred.

During AP, cardio-toxic pro-inflammatory cytokines and proteolytic enzymes are released. The conduction system is exquisitely sensitive to inflammatory insults, as localized inflammation may infiltrate and compress the His–Purkinje system, the AV and sinoatrial node. Furthermore, inflammatory cytokines can directly affect cardiac ion channel function [9]. Inappropriate activation and delayed elimination of trypsinogen cause trypsin accumulation, which impairs cardiac ATP-sensitive potassium channels [3]. The release of proteolytic enzymes is increased due to the “cardio-biliary reflex,” which exacerbates heart injury. As spinal nerves supplying the heart, gallbladder, and bile duct intersect, increased pressure from inflammation within the bile system can trigger vagal hyper-activation, resulting in coronary artery spasms and depression of the sinus node and AV conduction [10]. Finally, during AP myocardial interstitial oedema, cardiomyocyte hypoxia, and hypertrophy with stromal collagenization have been reported, possibly increasing the likelihood of AV conduction defects [11].

Marked SB has been associated with AP; however, it resolved with underlying disease treatment [12]. Furthermore, the literature describes a patient with chronic pancreatitis who experienced intermittent AVBs but had not received PM implantation [13]. Notably, a patient with panhypopituitarism requiring high-dose intravenous hydrocortisone developed profound SB, which, however, subsided with steroid discontinuation [14].

Our patient, instead, manifested a persistent advanced AVB that may have resulted from irreversible conduction system damage, due to the severe SIRS and high levels of circulating proteolytic enzymes (as expressed by peak lipase) and pro-inflammatory cytokines (as indirectly suggested by acute-phase hemodynamic condition).

To the best of our knowledge, panhypopituitarism-induced AP with consequent persistent advanced AVB has never been described. Consequently, no indications regarding the indication and timing for permanent PM implantation in this setting are available. Our patient’s rhythm has been monitored continuously throughout hospitalization, revealing the persistence of the pathological AVB. Having ruled out other possible reversible causes, a permanent PM was implanted. Post-discharge device interrogation revealed phases of appropriate intervention, suggesting that the rhythm disorder had not improved during the follow-up.

## Conclusions

Hypopituitarism may cause AP because of associated metabolic and endocrine impairments. AP, in turn, may provoke intracardiac conduction abnormalities. No similar cases of high-grade AVB in hypopituitarism-induced AP have been reported in the literature. While the indication and timing for permanent PM implantation are unclear in this setting, our case suggests that a PM is necessary if the AV block does not recover despite AP resolution. Further evidence to improve the management of rhythm disturbances in hypopituitarism patients who develop AP is required. Clinicians should be aware of this complex and relatively uncommon issue, which requires careful evaluation before permanent PM implantation.

## Data Availability

Data and material are available on reasonable request from the author.

## Abbreviations

AP: Acute pancreatitis
ECG: Electrocardiography/electrocardiogram
SB: Sinus bradycardia
AVB: Atrioventricular block
PM: Pacemaker
b.p.m.: Beats per minute
CT: Computed tomography
AIP: Acute interstitial pancreatitis
SIRS: Systemic inflammatory response syndrome
AV: Atrioventricular

## References

[1] Gapp J, Tariq A, Chandra S (2023) Acute pancreatitis. *StatPearls*. Published online February 9, 2023. https://www.ncbi.nlm.nih.gov/books/NBK482468/. Accessed 3 Dec 2023

[2] Pezzilli R, Barakat B, Billi P (1996) Electrocardiographic abnormalities in acute pancreatitis. Eur J Emerg Med 6(1):27–29 10340731

[3] Rubio-Tapia A, García-Leiva J, Asensio-Lafuente E, Robles-Díaz G, Vargas-Vorácková F (2005) Electrocardiographic abnormalities in patients with acute pancreatitis. J Clin Gastroenterol 39(9):815–818. 10.1097/01.MCG.0000177241.74838.57 16145345 10.1097/01.mcg.0000177241.74838.57

[4] Türkay C, Aydoǧan T, Karanfil A, Erkmen Uyar M, Selçoki Y, Kanbay M (2009) T-wave depletion and bradycardia possibly secondary to acute pancreatitis: review of the literature. Turk J Gastroenterol 20(4):295–297. 10.4318/TJG.2009.0031 20084577 10.4318/tjg.2009.0031

[5] Thoeni RF (2012) The revised Atlanta classification of acute pancreatitis: its importance for the radiologist and its effect on treatment. Radiology 262(3):751–764. 10.1148/RADIOL.11110947 22357880 10.1148/radiol.11110947

[6] Cambruzzi E, Lais PKL, Carvalho SL (2013) Pilocytic astrocytoma of sellar/suprasellar region determining endocrine manifestations. J Bras Patol Med Lab 49:139–142

[7] Alghamdi K, Albakri LA, Alotaibi Y, Alghamdi AH, Alaidarous S (2021) Coexistence of triphasic diabetes insipidus and cerebral salt wasting syndrome following extraction of sellar/suprasellar grade I pilocytic astrocytoma. Cureus. 10.7759/CUREUS.17661 34646703 10.7759/cureus.17661 PMC8487248

[8] Faienza MF, Delvecchio M, Indrio F, Francavilla R, Acquafredda A, Cavallo L (2009) Acute pancreatitis in a girl with panhypopituitarism due to craniopharyngioma on growth hormone treatment: a combination of risk factors. Horm Res 71(6):372–375. 10.1159/000223423 19506396 10.1159/000223423

[9] Lazzerini PE, Laghi-Pasini F, Boutjdir M, Capecchi PL (2019) Cardioimmunology of arrhythmias: the role of autoimmune and inflammatory cardiac channelopathies. Nat Rev Immunol 19(1):63–64. 10.1038/S41577-018-0098-Z 30552387 10.1038/s41577-018-0098-z

[10] Manning GW, Hall GE (1937) Vagus stimulation and the production of myocardial damage. Can Med Assoc J 37(4):314–318 20320744 PMC536140

[11] [Combined pathology of the pancreas and myocardium in myocardial infarction and acute destructive pancreatitis] - PubMed. https://pubmed.ncbi.nlm.nih.gov/9005827/. Accessed 3 Dec 2023 9005827

[12] Mathew TL, Gonzales G, Hoang L (2020) S1449 symptomatic bradycardia: an unusual presentation of acute pancreatitis. Am J Gastroenterol 115(1):S694–S694. 10.14309/01.AJG.0000707844.26131.86

[13] Chen-Scarabelli C, Saravolatz L, Scarabelli TM (2010) Intermittent AV block conduction abnormalities in the setting of acute pancreatitis. J Cardiol Cases. 10.1016/J.JCCASE.2010.05.010 30532815 10.1016/j.jccase.2010.05.010 PMC6265207

[14] Cimbek E, Kaya G, Ozturk M, Dilber E, Karagüzel G (2021) Corticosteroid-induced sinus bradycardia in a young boy with adrenal insufficiency and sepsis. Arch Argent Pediatr. 10.5546/AAP.2021.ENG.E353 34309317 10.5546/aap.2021.eng.e353

